# Endoscopic treatment of complete stripping of esophageal mucosal and submucosal tissue layers

**DOI:** 10.1055/a-2088-8607

**Published:** 2023-06-12

**Authors:** Francesca D’Errico, Maurizio Fazi, Jean-Loup Dumont, Gianfranco Donatelli

**Affiliations:** 1 Unité d’Endoscopie Interventionnelle, Hôpital Privé des Peupliers, Ramsay Générale de Santé, Paris, France; 2 Unit of Gastroenterology and Digestive Endoscopy, F. Miulli General Regional Hospital, Acquaviva delle Fonti, Italy; 3 Department of Clinical Medicine and Surgery, Federico II, University of Naples Naples, Italy

A 45-year-old woman with advanced lower esophageal cancer was palliatively treated with placement of a partially covered self-expandable metal stent (PCSEMS) and chemoradiotherapy. Removal of the stent was needed 3 months later because of migration and stent ingrowth. We did not use argon plasma coagulation to burn the overgrowth tissue 1 at the edges of the stent because the stent had migrated and it was not possible to pass the scope through it ( Fig. 1 ). Unfortunately, removal of the stent caused a stripping of the esophageal tissue layers. In detail, a 25-cm floppy “sleeve” of esophageal mucosa and submucosa was pulled out with the PCSEMS. The sleeve of tissue was intubated in order to visualize the de-epithelialized muscular layer ( Fig. 2 ); this layer was not perforated as confirmed later by an upper barium swallow study ( Fig. 3 ). The detached sleeve of esophageal tissue was pushed down again and a 12-cm fully covered SEMS and a nasoenteral feeding tube were inserted.

**Fig. 1:**
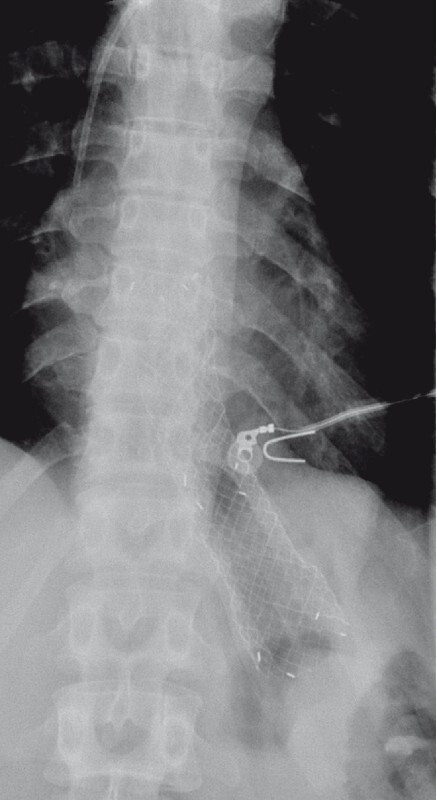
Radiograph showing the migrated self-expandable metal stent (SEMS). It was not possible to pass the endoscope through it.

**Fig. 2 a, b:**
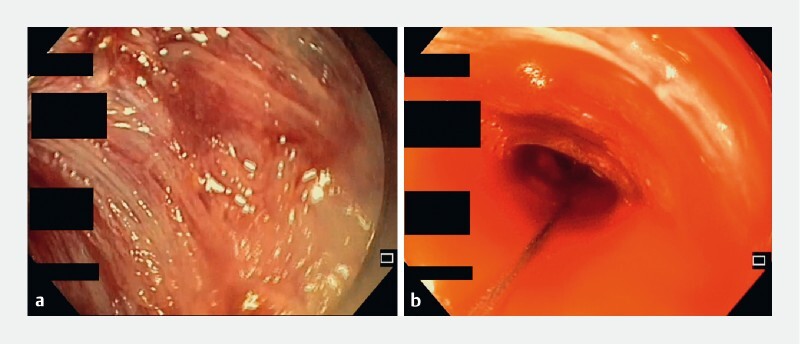
Upper endoscopy through the “sleeve” of dislocated esophageal mucosal and submucosal tissue, showing the de-epithelialized muscular layer.

**Fig. 3:**
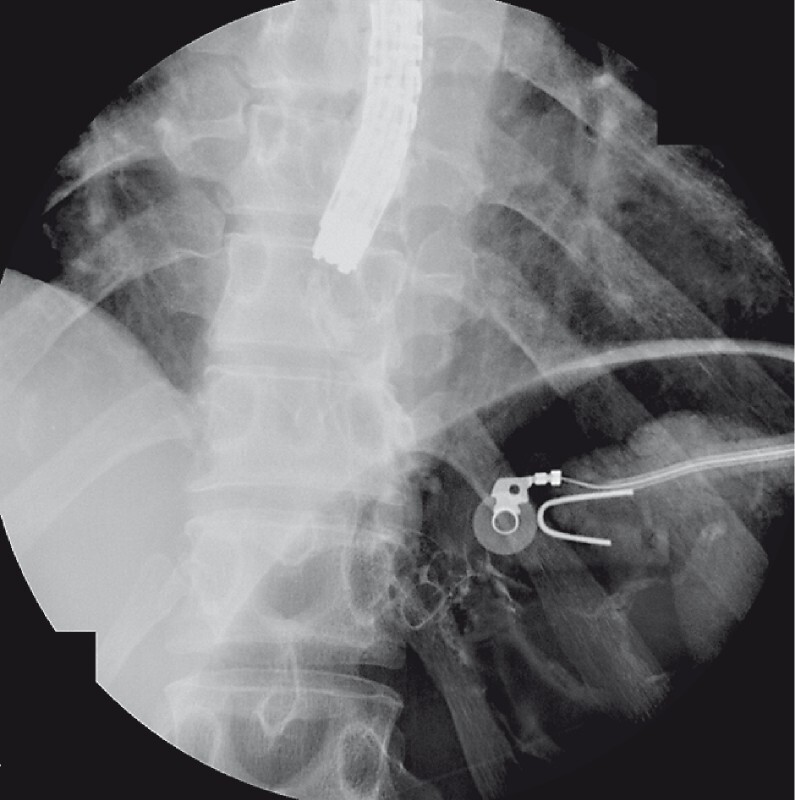
An upper barium swallow study showed no extravasation of contrast medium.

However, 4 hours later the patient presented with vomiting and exteriorization of the sleeve of esophageal tissue ( Fig. 4 , Video 1 ). After discussion with otorhinolaryngologists and surgeons, it was decided to endoscopically reposition the stripped esophageal tissue which would otherwise obstruct the patient’s airways and laryngeal inlet. The sleeve of tissue was grasped with two Kocher forceps ( Video 1 ) and the scope was inserted through it in order to remove the SEMS. Then, a Devière overtube (Wilson-Cook) was inserted, allowing the tissue sleeve to be pushed completely down, slowly and gently ( Fig. 5 ). A guidewire was passed and a long covered SEMS (17 cm) was left in place between the cervical and lower esophagus together with an NFT. Recovery was uneventful, with oral intake resumed 3 days later.

**Fig. 4:**
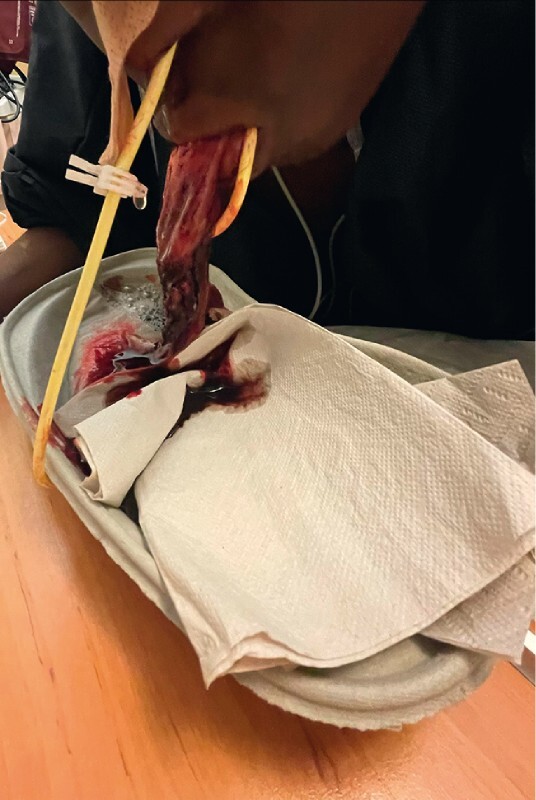
Exteriorization of the sleeve of esophageal tissue through the patient’s mouth.

**Video 1** A “sleeve” (here termed a “monchon”) of esophageal mucosal and submucosal tissue is pulled out during removal of a self-expanding metal stent (SEMS), and reinserted with the use of an overtube.


**Fig. 5:**
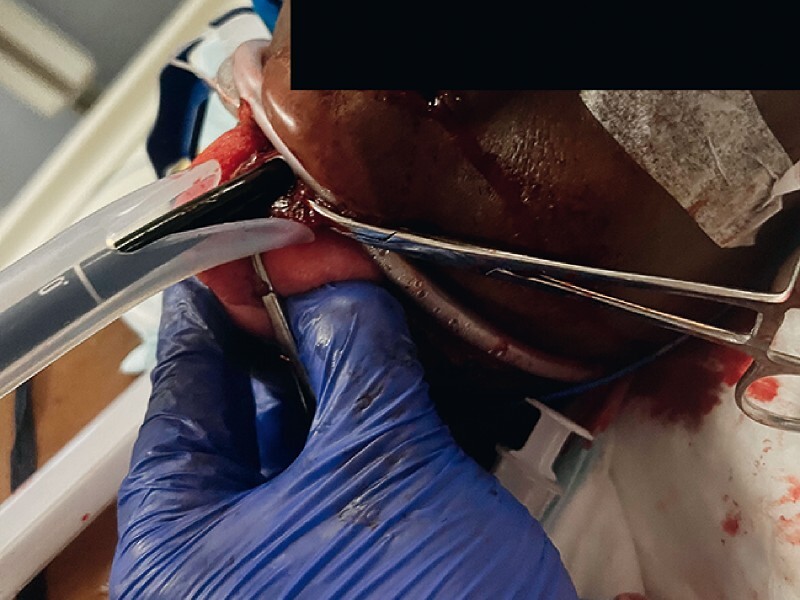
Insertion of the overtube over the scope allowed the sleeve of esophageal tissue to be pushed down slowly and gently.

We describe here an adverse event related to the use of PCSEMS, namely a stripping of superficial esophageal tissue layers, and completely endoscopic treatment of this complication. In conclusion, endoscopist experience and the availability of several endoscopic tools permitted the treatment of this nightmare adverse event in a patient who was not a candidate for surgery.

Endoscopy_UCTN_Code_CPL_1AH_2AD

## Notes

Endoscopy E-Videos

https://eref.thieme.de/e-videos
*E-Videos* is an open access online section of the journal *Endoscopy* , reporting on interesting cases and new techniques in gastroenterological endoscopy. All papers include a high-quality video and are published with a Creative Commons CC-BY license. Endoscopy E-Videos qualify for HINARI discounts and waivers and eligibility is automatically checked during the submission process. We grant 100% waivers to articles whose corresponding authors are based in Group A countries and 50% waivers to those who are based in Group B countries as classified by Research4Life (see: https://www.research4life.org/access/eligibility/ ). This section has its own submission website at https://mc.manuscriptcentral.com/e-videos
